# Borderline Personality Disorder Symptoms and Stressful Life Events: An Evaluation of Gene-Environment Interplay

**DOI:** 10.1016/j.bpsgos.2024.100390

**Published:** 2024-08-30

**Authors:** Vilde Sofie Arneberg, Vilde Sundsvold, Ludvig Daae Bjørndal, Eivind Ystrom

**Affiliations:** a The Office for Children, Youth and Family Affairs; City of Oslo; Oslo, Norway; b Akershus University Hospital, Oslo, Norway; c PROMENTA Research Center, Department of Psychology, University of Oslo, Oslo, Norway; d PsycGen Centre for Genetic Epidemiology and Mental Health, Norwegian Institute of Public Health, Oslo, Norway; e Centre for Research on Equality in Education, University of Oslo, Oslo, Norway

**Keywords:** Borderline personality disorder, Co-twin control, Elastic net regression, Stressful life events, Twin study

## Abstract

**Background:**

Borderline personality disorder (BPD) is associated with high rates of stressful life events (SLEs). It is unclear whether people who experience SLEs have more BPD symptoms after accounting for the effects of familial risk factors. Our aims in the current study were to 1) create a predictive model of BPD using stressors across age and contexts and 2) examine whether SLEs resulted in higher levels of BPD symptoms beyond the effects of genetic and environmental risk factors.

**Methods:**

The sample comprised 2801 twins from the Norwegian Institute of Public Health Twin Panel. Poisson regression was used to explore which SLEs predicted BPD symptoms. Elastic net penalized regression was conducted to develop a predictive model for SLEs and BPD symptoms. Co-twin control analyses were performed to differentiate between environmental and genetic factors.

**Results:**

SLEs experienced during childhood and adulthood were associated with BPD symptoms. A weighted polyevent risk score explained 22% of the total variation in symptoms. Shared environmental and heritable factors explained 31% and 47% of individual differences in BPD symptomatology, respectively. Measured SLEs explained 42% of the shared environmental risk for BPD. The predictive risk of SLEs for BPD was reduced when shared environmental and genetic factors were accounted for. However, SLEs increased risk of BPD symptoms beyond the effects of shared genetic and environmental factors.

**Conclusions:**

BPD symptomatology following SLEs cannot fully be explained by genetic and shared environmental factors. The SLE-BPD symptoms associations were primarily due to selection by family environments. It is important to identify familial factors that lead to both SLEs and BPD symptoms. SLEs remained associated with BPD symptoms beyond genetic and environmental confounding.

## Plain Language Summary

In this study, we sought to examine how stressful life events are associated with risk of borderline personality disorder. In a twin sample, we found that stressful life events were associated with risk of Borderline Personality Disorder symptoms in young adulthood when genetic and shared environmental confounding were accounted for. This could indicate that tressful life events in part have a causal influence on Borderline Personality Disorder symptoms.

Borderline personality disorder (BPD) is characterized by a pervasive pattern of instability in emotionality, behavior, cognition, and interpersonal relationships and is associated with psychosocial impairment and high mortality rates due to suicide (1,2). However, current knowledge about risk factors for BPD is limited, and there is a need to identify risk factors for BPD that have causal effects (3).

Multiple studies have found a high prevalence of stressful life events (SLEs) during childhood among individuals with BPD in clinical and community samples (4, 5, 6, 7, 8, 9, 10). In fact, SLEs have been found to be among the most significant environmental risk factors for BPD (3,11). One 2009 review also concluded that multiple studies have consistently identified associations between maltreatment in childhood and BPD development (12). In sum, the results of several studies have converged to suggest that SLE exposure in childhood is a risk factor for BPD.

Fewer studies have assessed associations between SLE exposure during adulthood and BPD risk (13). In a 10-year follow-up study of a clinical sample, McGowan *et al.* (14) found that patients with BPD reported a declining rate of abuse experiences across a 10-year period in adulthood. When examining BPD severity in a community sample, Conway *et al.* (15) did not find any associations between SLE exposure and subsequent BPD severity. In sum, the small number of studies that have examined associations between SLEs that occurred during adulthood and BPD symptoms underline the need for more research on this topic. Furthermore, several previous studies have examined the effects of single or clusters of SLEs on risk of BPD. Because a number of different SLEs may influence BPD, the influences of multiple life events should be examined.

BPD is moderately influenced by genetic factors, with heritability estimates (i.e., the proportion of variance explained by genetic variation) of around 40% from several twin studies (16). This implies that most of the variation in BPD symptoms is accounted for by environmental factors. While several studies suggest that SLEs are associated with increased risk of BPD symptoms, these associations could reflect confounding factors, such as genetics. Genetic factors also influence measures of the environment, including traumatic life experiences (17). Therefore, associations between environmental factors (such as SLEs) and outcomes (such as BPD) may partially reflect confounding by familial factors that influence both SLE exposure and BPD. Genetically informative designs, such as discordant twin designs, can account for genetic and shared environmental confounding and strengthen causal inference in observational studies (18).

In the current study, we sought to investigate the influences of SLE exposure during childhood and adulthood on risk of BPD symptoms in adults, with 2 primary aims: first, to use a measure of multiple SLEs to create an optimal predictive model of BPD, and second to examine whether SLE exposure would result in a higher level of BPD symptoms above and beyond the effects of shared genetic and environmental risk factors for BPD.

## Methods and Materials

### Sample

Participants were recruited from a study of psychiatric disorders in the Norwegian Institute of Public Health Twin Panel (19). We used data from 2 separate waves of data collection. All participants gave written informed consent to participate in the study. Approval was sought and received from the Regional Committee for Medical and Health Research Ethics (#2010/767).

#### Wave 1 Sample

Wave 1 (W1) includes questionnaire data collected in 1998 and psychiatric interviews conducted from 1999 to 2004 (20). In total, 2801 twins were interviewed. Zygosity was originally obtained with a combination of questionnaire data, blood samples, and microsatellite markers. Interviews were mostly conducted face to face, and most interviewers were senior clinical psychology graduate students. The W1 sample consisted of 1347 monozygotic (MZ) and 1454 dizygotic (DZ) twins. Participants were ages 19 to 36 years at W1 (mean age = 28.2). At W1, there were 902 MZ female participants, 530 DZ female participants, 445 MZ male participants, 238 DZ male participants, and 686 participants from opposite-sex DZ twin pairs.

#### Wave 2 Sample

A total of 2284 twins were reinterviewed by telephone from 2010 to 2011, comprising the wave 2 (W2) sample. A total of 2299 twins also responded to a questionnaire at W2, which also assessed experiences of SLEs (only individuals with complete data for both waves of interviews and the SLE questionnaire were included in analyses). Participants were ages 30 to 44 years at W2 (mean age = 37.8). At W2, there were 778 MZ female participants, 418 DZ female participants, 357 MZ male participants, 188 DZ male participants, and 543 participants from opposite-sex DZ twin pairs.

### Measures

The SLE assessments were based on a previously used questionnaire measure (21). The participants were asked to report their age at the time when the SLE occurred if they had experienced a given SLE. In total, 18 SLEs were measured. The questionnaire was only administered at W2. SLEs experienced during childhood were defined as SLEs that occurred until participants were 18 years of age, whereas SLEs experienced during adulthood comprised events that occurred after age 18.

The Norwegian version of the Structured Interview for DSM-IV Personality assessing the DSM-IV Axis II was used to assess personality disorders. The Structured Interview for DSM-IV Personality is a semistructured interview that assesses all DSM-IV Axis II personality disorders, with the criteria scored on a 4-point scale from 0 to 3. To put the symptom count on the scale of the DSM-IV diagnosis, we used the following 4-point scale in our analyses: not present = 0, subthreshold = 0.5, present = 1, and strongly present = 1.5 (20,22). The interview assesses behaviors, cognitions, and feelings that predominated for most of the past 5 years because that is considered to be representative of long-term personality (23).

### Statistical Analyses

#### Generalized Linear Model

After examining initial descriptive statistics, we applied negative binomial regression models for Poisson distributions to examine which SLEs could predict BPD symptoms. This model is appropriate when the dependent variable is a count of the number of times an event occurs—in this case, number of BPD symptoms. The negative binomial model is chosen when it is assumed that events (i.e., criteria) are dependent, which leads to an overdispersion when the variance is larger than the mean.

For these models, BPD symptom was the dependent variable, and SLEs experienced during childhood and adulthood were independent variables. Standard errors were corrected for dependence in the data due to twin pairing using a sandwich estimator. The analysis explored the relative risk (RR) of having BPD symptoms if each SLE had been experienced (controlling for other SLEs), as well as the RR of having BPD symptoms if any SLE had been experienced during childhood or adulthood. Two separate grouped variables were created, with one comprising all SLEs experienced during childhood and the other comprising all SLEs experienced during adulthood.

#### Elastic Net Regression

Elastic net is a linear model–based methodology that shrinks regression coefficients toward zero and automatically selects predictors with nonzero coefficients (24). Elastic net is a regularization method for regression and classification models that comprises the least absolute shrinkage and selection operator penalty (L1) and the ridge penalty (L2). Regularization adds a hyperparameter lambda to regression models. Regression with elastic net regularization minimizes the ordinary sum of squared residuals while imposing additional penalty terms on regression parameters. This helps reduce problems of traditional variable selection methodologies related to poor prediction performance in external datasets due to overfitting. Elastic net shows advantages for variable selection as well as handling of highly correlated variables and has been demonstrated to fit large numbers of predictors (24). The elastic net method tests complete models, but at the cost of sampling distributions for individual parameters. In sum, elastic net regression serves as an approach for selecting the most parsimonious and best-fitting model when investigating many highly correlated factors for an outcome.

The 18 SLEs were divided into 2 clusters that contained all 18 SLEs in childhood and in adulthood, which made a total of 36 SLEs. The elastic net method was used to create a predictive polyevent risk score for the effect of SLEs on BPD symptoms. The polyevent risk score was constructed as the weighted sum of the SLEs that predicted number of BPD symptoms. The model selected variables that were strongly correlated with BPD symptoms and regulated noncorrelated variables to zero. The chosen model was the model with the lowest cross-validation error.

#### Co-Twin Control Analysis

Discordant twin designs compare twins who differ in exposure to the risk factor so that the unexposed twin serves as a control for the exposed co-twin (18). Thus, the co-twin control design allows for comparing risk of mental disorders within twin pairs in which one twin has experienced a stressful event and the other has not. This increases control over shared genetic and environmental confounders, which are fully shared by MZ twins and partially shared by DZ twins. In the current study, the co-twin control analysis allowed for adjusting for familial confounders of observed associations between SLEs and BPD symptoms.

First, we tested for overdispersion to allow the use of a Poisson regression. After accounting for random effects at the individual level, we found no overdispersion at the within-subject level (i.e., number of BPD symptom criteria after twin pair and time-invariant individual level risk was accounted for). Next, to estimate associations between SLEs and BPD, we ran a generalized linear mixed model with a Poisson distribution including BPD symptom as the dependent variable and sex and wave as fixed effects (i.e., baseline model). Estimating a second model according to Rabe-Hesketh *et al.* (25) was carried out where the fixed effects included between-twin pair effects (i.e., additive genetic effects [A] = 1.0 for MZ twins / 0.5 for DZ twins and shared environmental effects [C]), and an within twin pair effect for DZ twins of A = 0.5. MZ twins are 100% identical by descent, and therefore they share 1.0A. DZ twins are 50% identical by descent on average, and therefore they share 0.5A. Both MZ and DZ twins share environments that have effects on outcomes in both twins 1.0C. The remaining nonfamilial variance is attributed to nonshared environmental effects (E). All twin pairs shared residual random between effects of 0.5A and 1C. MZ twins shared an additional random between effect of 0.5A to account for being 50% more identical by descent compared with DZ twins. To model residual genetic effects due to genetic recombination, DZ twins had a 0.5A random effect at the within-subject level. Finally, the 2 models were compared to examine the influence of A, C, and E effects on the association between SLEs and BPD symptoms. Model fit was evaluated using Akaike information criterion (AIC) (26). All analyses were carried out in Stata MP 16.

## Results

### Descriptive Statistics

In Table 1, we present the number of participating twins by zygosity and gender. The SLEs with highest prevalence in the sample were having divorced or separated, having experienced parental mental illness or alcohol problems as a child, and having experienced parental divorce or separation as a child. Prevalences and RRs associated with SLE exposure for all SLEs are reported in Table 2.

**Table 1:** Number of Participants at Each Wave of Data Collection

	Wave 1	Wave 2
Monozygotic Twin		
Female	902	778
Male	445	357
Dizygotic Twin		
Female	530	418
Male	238	188
Opposite Sex	686	543

**Table 2:** Rates of Stressful Life Events and Relative Risk of Borderline Personality Disorder Symptoms Aaccording to Stressful Life Event Exposure (*n* = 5075 Measures of BPD)

Stressful Life Events	Childhood or Adulthood	Presence of BPD Symptoms, *n* = 2151 Measures of BPD (%)	No BPD Symptoms, *n* = 2924 Measures of BPD (%)	RR for BPD Symptoms (95% CI)
SLE1: Life-Threatening Illness	Childhood	40 (1.86%)	48 (1.64%)	1.32 (0.92–1.89)
	Adulthood	54 (2.50%)	46 (1.57%)	1.61 (1.20–2.15)
SLE2: Life-Threatening Accident	Childhood	44 (2.04%)	45 (1.54%)	1.63 (1.08–2.46)
	Adulthood	82 (3.81%)	76 (2.60%)	1.18 (0.93–1.49)
SLE3: Actively Participated in War/Combat	Childhood	0 (0%)	0 (0%)	1 (omitted)
	Adulthood	23 (1.07%)	28 (0.96%)	0.91 (0.60–1.49)
SLE4: Witnessed Anyone Be Badly Injured or Killed	Childhood	40 (1.86%)	54 (1.85%)	1.38 (0.95–1.99)
	Adulthood	107 (4.97%)	131 (4.48%)	1.04 (0.82–1.32)
SLE5: Threatened With a Weapon, Held Captive, or Kidnapped	Childhood	26 (1.21%)	29 (0.99%)	1.52 (1.04–2.23)
	Adulthood	110 (5.11%)	85 (2.91%)	1.56 (1.24–1.95)
SLE6: Experienced Fire, Flooding, or Natural Disaster	Childhood	46 (2.14%)	27 (0.92%)	1.67 (1.13–2.46)
	Adulthood	60 (2.79%)	76 (2.60%)	1.29 (0.87–1.89)
SLE7: Rape	Childhood	55 (2.56%)	34 (1.16%)	2.30 (1.69–3.13)
	Adulthood	51 (2.37%)	23 (0.79%)	2.71 (2.04–3.59)
SLE8: Sexual Abuse	Childhood	162 (7.53%)	110 (3.76%)	1.84 (1.52–2.22)
	Adulthood	32 (1.49%)	20 (0.68%)	2.64 (1.77–3.94)
SLE9: Otherwise Physically Attacked or Assaulted	Childhood	120 (5.58%)	65 (2.22%)	2.10 (1.66–2.67)
	Adulthood	169 (7.86%)	136 (4.65%)	1.92 (1.60–2.30)
SLE10: Otherwise Physically Abused as a Child	Childhood	90 (4.18%)	46 (1.57%)	2.60 (2.09–3.22)
	Adulthood	0 (0%)	0 (0%)	1 (omitted)
SLE11: Otherwise Mistreated as a Child	Childhood	79 (3.67%)	31 (1.06%)	3.28 (2.60–4.14)
	Adulthood	0 (0%)	0 (0%)	1 (omitted)
SLE12: Parental Mental Illness or Alcohol Problems as a Child	Childhood	298 (13.85%)	218 (7.46%)	1.91 (1.61–2.26)
	Adulthood	6 (0.79%)	5 (0.17%)	1.17 (0.57–2.40)
SLE13: Parental Divorce or Separation as a Child	Childhood	362 (16.83%)	373 (12.76%)	1.48 (1.27–1.72)
	Adulthood	28 (1.30%)	42 (1.44%)	1.35 (0.69–2.62)
SLE14: Having Divorced or Separated	Childhood	5 (0.23%)	3 (0.10%)	2.89 (1.20–6.95)
	Adulthood	404 (18.78%)	453 (15.49%)	1.26 (1.12–1.43)
SLE15: Long-Term Financial Difficulties	Childhood	10 (0.46%)	4 (0.14%)	1.76 (0.94–3.28)
	Adulthood	118 (5.48%)	72 (2.46%)	2.20 (1.84–2.64)
SLE16: Unemployment for More Than 6 Months	Childhood	12 (0.56%)	6 (0.21%)	2.19 (0.94–5.11)
	Adulthood	134 (6.23%)	126 (4.31%)	1.54 (1.26–1.87)
SLE17: Major and Lasting Conflict With Close Person	Childhood	27 (1.25%)	13 (0.44%)	2.80 (1.93–4.05)
	Adulthood	148 (6.88%)	148 (5.06%)	1.69 (1.36–2.10)
SLE18: Other Serious Life Event	Childhood	79 (3.67%)	62 (2.12%)	2.05 (1.53–2.74)
	Adulthood	154 (7.16%)	200 (6.84%)	1.31 (1.09–1.59)

BPD, borderline personality disorder; RR, relative risk; SLE, stressful life event.

### Poisson Regression Analyses

We examined associations between both individual SLEs and clustered SLEs using negative binomial Poisson regression analyses. Multiple individual SLEs were associated with increased risk of BPD symptoms. The clustered variable comprising childhood SLEs was significantly associated with an increased risk of BPD symptoms (RR = 1.38; 95% CI, 1.35–1.42), as was SLEs in adulthood (RR = 1.24; 95% CI, 1.18–1.31).

#### Elastic Net Regression

Next, we conducted elastic net regression with a Poisson distribution to create a predictive model for BPD symptoms. These results are shown in Table 3. The elastic net revealed 25 SLEs uniquely related to BPD symptoms. Three different α values were tested (α = 1, α = 0.75, and α = 0.5), and α = 0.5 was chosen to specify elastic net instead of least absolute shrinkage and selection operator (α = 1) or ridge regression (α = 0), using 10-fold cross-validation. This α was chosen due to the optimal ƛ value, which in this case was ƛ = 0.02.

**Table 3:** Associations From Best-Fitting Elastic Net Regression Predicting Borderline Personality Disorder Symptoms by Stressful Life Events

Stressful Life Events	Childhood or Adulthood	RR of BPD Symptoms^a^
SLE1: Life-Threatening Illness	Childhood	–
	Adulthood	1.23
SLE2: Life-Threatening Accident	Childhood	–
	Adulthood	1.17
SLE3: Actively Participated in War/Combat	Childhood	–
	Adulthood	–
SLE4: Witnessed Anyone Be Badly Injured or Killed	Childhood:	1.17
	Adulthood	–
SLE5: Threatened With a Weapon, Held Captive, or Kidnapped	Childhood	0.73
	Adulthood	1.21
SLE6: Experienced Fire, Flooding, or Natural Disaster	Childhood	1.19
	Adulthood	–
SLE7: Rape	Childhood	1.46
	Adulthood	1.59
SLE8: Sexual Abuse	Childhood	1.15
	Adulthood	1.08
SLE9: Otherwise Physically Attacked or Assaulted	Childhood	1.40
	Adulthood	1.56
SLE10: Otherwise Physically Abused as a Child	Childhood	1.19
	Adulthood	–
SLE11: Otherwise Mistreated as a Child	Childhood	1.88
	Adulthood	–
SLE12: Parental Mental Illness or Alcohol Problems as a Child	Childhood	1.44
	Adulthood	–
SLE13: Parental Divorce or Separation as a Child	Childhood	1.15
	Adulthood	0.69
SLE14: Having Divorced or Separated	Childhood	–
	Adulthood	1.11
SLE15: Long-Term Financial Difficulties	Childhood	1.24
	Adulthood	1.19
SLE16: Unemployment for More Than 6 Months	Childhood	–
	Adulthood	1.23
SLE17: Major and Lasting Conflict With Close Person	Childhood	1.12
	Adulthood	1.32
SLE18: Other Serious Life Event	Childhood	1.33
	Adulthood	1.18

BPD, borderline personality disorder; RR, relative risk; SLE, stressful life event.

a “–” denotes an effect which was set to 0 in the elastic net regression model. The fixed effect of wave was 0.65. The fixed effect of sex was 1.05.

We established a polyevent risk score, which was a predictive score for BPD symptoms based on the SLEs selected in the elastic net model (Table 3). The predicted number of BPD criteria ranged from 0.32 to 7.64. The risk score was also correlated with the initial BPD symptom score measured at a single time point (*r* = 0.33), indicating that the model was moderately predictive of BPD symptoms. The predicted number of BPD symptoms had a variance of 0.13, a mean of 0.65, and a median of 0.61.

#### Co-Twin Control Analysis

We carried out a multilevel Poisson regression model and found the effect within MZ pairs to be RR = 1.33 and the effect within DZ pairs to be RR = 1.29. The additional effect of DZ pairs on the between level was RR = 1.03 and thus close to no effect. All similarity was assigned to shared environment on the between-subjects level. The results of the multilevel Poisson model are shown in Table 4.

**Table 4:** Biometric Co-Twin Control: Effect Within the Whole Sample, DZ Pairs, and MZ Pairs

Sample	Poisson Regression		Probit Regression	
	RR	95% CI	β	95% CI
Whole Sample	3.14	2.52–3.91	1.51	1.22–1.79
Within Dizygotic Twin Pairs	1.29	1.06–1.56	0.41	0.17–0.65
Within Monozygotic Twin Pairs	1.33	1.10–1.60	0.44	0.21–0.68

RR, relative risk.

The AIC was lower for the ACE model (AIC = 8837) than the model without a C effect (i.e., the AE model) (AIC = 8976), indicating that the ACE model comprising both additive genetic and shared environmental effects was the better-fitting model. The ACE models are presented in Table 5. The unstandardized coefficient was used as a basis for comparing the effects of A, C, and E. In the baseline model (adjusting for sex and wave), these effects were estimated to be A = 0.36, C = 0.24, and E = 0.17. In standardized metrics, this corresponds to a heritability of 47%, a shared environmental effect of 31%, and a nonshared environmental effect of 22%. When the polyevent risk score was included, the effects in the ACE model were A = 0.30, C = 0.14, and E = 0.16, which postulates a heritability of 60%, a shared environmental effect of 23%, and a heritability of 27% in a theoretical selected population with no experiences of the life events evaluated in this study. We found that the shared environmental effect estimate decreased the most with 41.7% (−0.1), followed by the genetic effect estimate with a decrease of 16.7% (−0.06). The effect of nonshared environmental factors did not decrease remarkably (5.9% or −0.01). Summing up the explained variance at the 3 levels, the polyevent risk score explained 22% of the total stable, or time-invariant, variance in BPD symptoms.

**Table 5:** Random Effects Estimates From Linear Mixed Models of Borderline Personality Disorder Symptoms Predicted by Stressful Life Events

	Symptom Count				Ordinal Liability-Threshold			
	Poisson Distribution				Probit Normal Distriubtion			
	Baseline		SLEs Residualized		Baseline		SLEs Residualized	
	Estimate	%	Estimate	%	Estimate	%	Estimate	%
Additive Genetic, A	0.36	47%	0.30	50%	0.44	59%	0.36	66%
Shared Environmental, C	0.24	31%	0.14	23%	0.14	19%	0.05	10%
Nonshared Environmental, E	0.17	22%	0.16	27%	0.16	22%	0.14	25%
Residual Variance	0.59		0.57		1.00		1.00	
Total Variance	1.36		1.17		1.74		1.55	

The standardization of the A, C, and E random effects were based on the total variance of A + C + E to give percentage of variance explained.

SLE, stressful life event.

## Discussion

The aims of this study were to investigate associations between SLEs and BPD symptoms and to what extent these associations are accounted for by familial genetic and environmental factors. We found that 47% of variance in BPD symptoms was explained by genetic factors, 31% was explained by family shared environmental factors, and 22% was explained by nonshared environmental factors. Both individual and clustered SLEs were associated with increased risk of BPD symptoms. The polyevent risk score was associated with increased risk of BPD symptoms in the total sample. The SLE-BPD associations were reduced in DZ and MZ twins, which is indicative of partial confounding by shared environmental factors. However, the SLEs accounted for 41.7% of the shared environmental risk for BPD symptoms, indicating that SLEs shared by siblings are of considerable importance for BPD.

Thirteen SLEs experienced during childhood were associated with increased risk of BPD symptoms in the elastic net regression model. Having experienced rape, having experienced physical abuse as a child, and having been otherwise mistreated as a child were all associated with increased risk of BPD symptoms. These results are consistent with results of several studies that have indicated that sexual abuse, emotional abuse, and neglect are associated with BPD (8,9,12). This is also consistent with previous studies that have suggested that SLEs experienced early in life and childhood adversity are highly prevalent in people with personality disorders, including BPD (4,5,7). The risk of BPD symptoms was more strongly associated with SLEs experienced during childhood than during adulthood.

Twelve SLEs in adulthood were associated with BPD symptoms in the elastic net regression model, including having divorced or separated, having experienced a major and lasting conflict with a close person, having experienced rape, and having experienced long-term economic issues. Having experienced rape was most strongly associated with increased risk of BPD symptoms.

A polyevent risk score, created using elastic net regression, was moderately predictive of BPD symptom risk at a single time point. This model identified multiple SLEs in childhood and adulthood that were associated with risk for BPD symptoms, such as witnessing and/or experiencing life-threatening experiences; experiencing an unsafe and unpredictable childhood environment with sexual and physical abuse, mistreatment, and violence and having a parent who suffered from mental illness; and having experienced economic issues.

The co-twin control analyses supported a multifactorial causal explanation of the relationship between SLE exposure and BPD symptoms because the effect of exposure was reduced when the effects of shared environmental and genetic factors were controlled for. Importantly, the polyevent SLE score explained 42% of the environmental risk shared by siblings. Admittedly, co-twin control designs only have the power to evaluate events that siblings can be reasonably discordant on. However, the considerable reduction in explanation of shared environmental risk for BPD symptomatology shows that multiple measurable events shared by siblings who grew up in the same family can potentially have a large impact on BPD in young adulthood. Our results are not consistent with previous discordant twin studies that examined the SLE-BPD relationship, which have found little to no evidence of a causal effect of SLEs on BPD symptoms (27,28).

When we adjusted for the measures of the environment included in the polyevent risk score, the variance explained by shared environmental factors decreased the most. It appears to serve as a confounder between SLEs and BPD symptoms, meaning that it seems to be something in the effective environment that contributes to both the experience of SLEs and the development of BPD symptoms (Turkheimer & Waldron, 2000). The pattern of confounding was indicative of partial and not complete confounding (i.e., the effect decreased but was not completely absent). One previous study also found that the association between childhood trauma and personality disorder was accounted for by familial factors, but low statistical power did not allow for examining DZ and MZ pairs separately (27). Partial confounding by shared environmental factors was not found by Bornovalova *et al.* (28), who found that the association was better explained by genetic factors. We note that some shared environmental effects remain unaccounted for by the SLEs assessed in our study, which implies that the influence of the effective environment on BPD symptoms was not fully explained by the measures of SLEs included.

Genetic effects decreased a small amount when we adjusted for the SLEs included in the polyevent risk score, indicating that SLEs were linked to some genetic factors for BPD symptoms. These putative causal effects constitute mediated genetic effects (i.e., SLEs explain genetic variance in BPD through a causal path because SLEs are influenced by genetic factors). This is consistent with the findings of one previous study suggesting that the SLE-BPD association is explained by genetic factors (28). However, the genetic risk for BPD symptoms seemed to be primarily unaccounted for by the SLEs in the polyevent risk score. This means that any genome-wide association study of BPD symptoms will find alleles that confer risk for SLEs only to a moderate degree (i.e., estimated at 16.7% in the current study) and find alleles that do not have a mediated effect on BPD through SLEs to a great degree.

### Strengths and Limitations

The current study has several strengths. First, we leveraged data from a genetically informative and large sample from the Norwegian Twin Registry. Second, BPD symptoms were assessed using clinical interviews considered to be representative of long-term personality (23) at 2 separate time points nearly 10 years apart. Third, we examined the effects of multiple SLEs and not individual SLEs, as has been done in many previous studies. Fourth, we used a regularization regression method to ensure model sparsity. Thus, we evaluated the best-fitting model to our dataset instead of individual *p* values, which is an approach with limitations that are well-established (29). We used symptom criteria including subthreshold symptoms, which are likely to capture more individuals with BPD symptoms than using clinical diagnoses only.

Our study also has several limitations. First, the data on SLE exposure were based on retrospective report. Although the validity of reporting was possibly enhanced by the use of a semistructured interview approach, there might still have been limitations in reporting due to limitations in memory, repression, denial, or deliberate over- and/or underreporting. Furthermore, it has been found that prospectively and retrospectively reported SLEs identify substantially non-overlapping groups of individuals. Thus, the extent to which associations between SLEs and BPD symptom risk are similar when prospective SLE measures are used should be examined. Furthermore, although it was required that events occurred before the BPD assessment, we cannot exclude the possibility that some reported events could be consequences of BPD symptoms. The multiple assessments of BPD symptoms also contribute to difficulties in identifying SLEs that may cause BPD symptoms or reflect their consequences. Second, we cannot rule out the possibility of reverse causality in the observed associations between SLEs and BPD symptoms, i.e., the possibility that instead of SLEs in adulthood exerting a causal influence on BPD symptom risk, BPD symptoms could exert a causal influence on SLE exposure risk. Third, elastic net regression selects the most predictive model at the cost of individual parameters, meaning that there is no meaningful way to estimate confidence intervals for individual predictors in the model. Fourth, although the discordant twin design controls for variables shared by the twins, it is vulnerable to the influence of unmeasured and unshared confounding variables (18). Fifth, our sample consisted of Norwegian twins only, and the extent to which our results are generalizable to other populations is unclear. Sixth, because symptoms were required to have predominated for a period of at least 5 years, we cannot exclude the possibility that we underestimated symptom prevalence in younger participants. Finally, previous studies have reported a modest selection effect in this sample toward better mental and somatic health (20).

### Conclusions

We found that SLEs experienced during both childhood and adulthood were associated with increased risk of BPD symptoms. Using elastic net regression, we found that a polyevent risk score was moderately predictive of risk of BPD symptoms. Co-twin control analyses indicated that the associations between SLE exposure and BPD symptoms were partially confounded by shared environment. The measures of SLEs shared by siblings who grew up in the same family accounted for a large proportion of the shared environmental risk for BPD symptoms. Our results point to there being something in the shared family environment that causes both the SLEs and the development of BPD symptoms. Therefore, it is important to limit the effect of, or prevent, familial environmental background factors that cause both SLEs and BPD symptoms.

## Supplementary Material

Key Resources Table
