# Supplementary material for: Borderline Personality Disorder Symptoms and Stressful Life Events: An Evaluation of Gene-Environment Interplay
Source: Biol Psychiatry Glob Open Sci. 2024 Aug 30;4(6):100390. doi: 10.1016/j.bpsgos.2024.100390 (PMC11740797; doi:10.1016/j.bpsgos.2024.100390)
Supplement: Supplementary Table 1 [file mmc2.pdf]

SUPPLEMENTARY INFORMATION

Borderline Personality Disorder Symptoms and Stressful Life Events: An Evaluation of Gene-Environment Interplay

Arneberg *et al.*

Supplementary table 1. Cross-twin correlations for observed borderline personality disorder symptoms and SLE predicted symptoms.

		Twin 1		Twin 2	
		Observed	SLE predicted	Observed	SLE predicted
Twin 1	Observed	1.00	.38	.21	.22
	SLE predicted	.28	1.00	.17	.50
Twin 2	Observed	.28	.25	1.00	.33
	SLE predicted	.23	.54	.29	1.00

Note. Correlations for dizygotic twins above the diagonal and correlations for monozygotic twins below the diagonal.
